# Genetic Variants and Increased Expression of *Parascaris equorum* P-glycoprotein-11 in Populations with Decreased Ivermectin Susceptibility

**DOI:** 10.1371/journal.pone.0061635

**Published:** 2013-04-24

**Authors:** I. Jana I. Janssen, Jürgen Krücken, Janina Demeler, Marta Basiaga, Sławomir Kornaś, Georg von Samson-Himmelstjerna

**Affiliations:** 1 Institute for Parasitology and Tropical Veterinary Medicine, Freie Universität Berlin, Germany; 2 Department of Zoology and Ecology, University of Agriculture in Krakow, Poland; Auburn University, United States of America

## Abstract

Macrocyclic lactones (MLs) represent the major drug class for control of parasitic infections in humans and animals. However, recently reports of treatment failures became more frequent. In addition to human and ruminant parasitic nematodes this also is the case for the horse-nematode *Parascaris equorum*. Nevertheless, to date the molecular basis of ML resistance is still not understood. Unspecific resistance mechanisms involving transporters such as P-glycoproteins (Pgps) are expected to contribute to ML resistance in nematodes. Here, complete sequences of two *P. equorum* Pgps were cloned and identified as orthologs of *Caenorhabditis elegans* Ppg-11 and an unnamed *Caenorhabditis briggsae* Pgp designated as Pgp-16 using phylogenetic analysis. Quantitative real-time PCR was used to compare expression between tissues. Significantly higher *Peq*Pgp-11 expression was found in the gut for both genders, whereas for *Peq*Pgp-16 the body wall was identified as predominant expression site. Furthermore, Pgps were analyzed regarding their participation in resistance development. Using SeqDoC analyses, Pgp-sequences of *P. equorum* populations with different ML susceptibility were compared. This approach revealed three single nucleotide polymorphisms (SNPs) causing missense mutations in the *Peq*Pgp-11 sequence which correlated with decreased ML susceptibility. However, no resistance associated differences in mRNA expression levels were detected between embryonated eggs of these populations. In contrast, comparison of two pre-adult groups with different ivermectin (IVM) susceptibility revealed the presence of the three SNPs and in addition statistically significant *Peq*Pgp-11 overexpression in the group of worms with reduced susceptibility. These results indicate that Pgp-11 might be involved in IVM resistance in *P*. *equorum* as it shows increased expression in an IVM exposed life-cycle stage of an IVM resistant population as well as occurrence of putatively resistance associated SNPs in populations with reduced IVM susceptibility. These SNPs are promising diagnostic candidates for detection of ML resistance with potential also for other parasitic nematode species.

## Introduction

For decades macrocyclic lactones (MLs) have been used for the treatment and control of infections with endoparasitic nematodes and ectoparasitic arthropods in human and veterinary medicine [1]. Due to their extensive use, ML resistance has evolved in several parasites, as it also has been described for the two other major families of broad-spectrum anthelmintics routinely used in livestock, the benzimidazoles (BZ) and the agonists of the nicotinic acetylcholine receptor such as the tetrahydropyrimidine pyrantel and the imidazothiazole levamisole [2], [3], [4]. Even though the prevalence of ML resistant nematode populations increases, the resistance mechanisms remain largely unclear. Several possible mechanisms are discussed, including the activity of ATP-binding-cassette (ABC)-transporters, in particular P-glycoproteins (Pgps) [5]. Pgps are located in the apical cell-membrane, act as ATP-dependent transporters for hydrophobic xenobiotics such as anthelmintics and decrease the concentration of the drugs at their target sites [6]. This effect finally prohibits an efficient treatment.

A correlation between Pgps and resistance to both BZs and MLs has already been described for some nematodes [7], [8], [9], [10]. For *Caenorhabditis elegans*, 14 Pgp genes and one pseudogene have been published [11], [12] or annotated in Wormbase and use of a Pgp-3 deficient strain demonstrated that this protein decreases sensitivity to colchicine and chloroquine [7]. In contrast, the loss of Pgp-1 results in hypersensitivity to heavy metals [13]. More recently, an overexpression of five Pgps was demonstrated in an ivermectin (IVM) resistant *C. elegans* strain [14]. Furthermore, eight Pgps were reported in *Brugia malayi* [15], nine full or partial sequences in *Haemonchus contortus* [10], [16], [17], [18], at least two Pgp cDNAs in cyathostomins [19], eleven partial sequences in *Teladorsagia circumcincta* [20] and two full-length Pgp-encoding sequences in *Onchocerca volvulus* [17], [21]. However, there are currently no database entries for *Parascaris equorum* Pgps although there are several *P. equorum* populations known to show reduced susceptibility or even resistance against IVM as reported from various countries in Europe and the Americas [22].

Due to its global distribution, high prevalence and severe pathogenicity, the nematode *P. equorum* is one of the most important parasitic pathogens of equines. In particular in foals and yearlings parascarosis can cause severe illness and even death. Infected horses can show several clinical signs, depending on the worm burden and ranging from nasal discharge and coughing, to a bad general condition and ceased growth, up to lethal intestinal obstruction and/or rupture. Treatment is generally carried out with BZs, tetrahydropyrimidines or MLs. In this context, *P. equorum* takes an exceptional position as it is the dose-limiting species for all currently available broad-spectrum anthelmintics in horses except for the tetrahydropyrimidines [23]. Moreover, due its high pathogenicity for foals and yearlings, many horse breeders deworm this age group more than six times a year [24]. Therefore, it is not surprising that decreased susceptibility to anthelmintics in *P. equorum* is rapidly spreading and currently has become a key health concern for foals and young horses on many stud farms. This emphasizes the importance to decipher resistance mechanisms and to identify markers which can be used to detect developing resistance at an early stage. However, the number of routine molecular diagnostic tests for detection of anthelmintic resistance is restricted to the BZs, where resistance markers could already be identified and evaluated. Three single nucleotide polymorphisms (SNPs) which cause changes at codons 167, 198 or 200 in the β-tubulin isotype 1 gene are present in many BZ-resistant nematode populations [5]. In contrast, results for the MLs are more diverse: studies of the *T. circumcincta* and *H. contortus* glutamate-gated chloride channel (GluCl) subunits revealed no changes of the target genes which might be responsible for anthelmintic resistance [25], [26]. On the other hand, mutations of the GluCl-subunits of *Cooperia oncophora* have been shown to cause a significant decrease in sensitivity to glutamate and IVM [27], although the identified SNPs have not yet been reported in any IVM resistant field isolates since then. Results appear to be similarly variable for the Pgps where a high frequency of polymorphisms in *pgp-9* of *T. circumcincta* was demonstrated but no resistance associated coding SNPs for the investigated region were identified [20]. It has also been shown that the restriction patterns of *H. contortus pgp-2* differ between IVM susceptible and IVM resistant populations [10]. Additionally, slightly increased pgp-2 and pgp-9 mRNA expression levels were demonstrated in resistant isolates of *H. contortus* [10], [28] as well as *T. circumcincta* pgp-9 [20] and *C. elegans* pgp-1 and pgp-2 [29]. In contrast, a decreased expression was shown for pgp-1 in *H. contortus*.

The techniques to detect ML resistance in *P. equorum* populations are very limited. Since there are no laboratory hosts for *P. equorum* and *in vitro* culture is very labor intensive and only possible for a short time, molecular resistance markers would help to develop a particularly attractive diagnostic tool. Currently, the only option to diagnose resistance for this parasite is the fecal egg count reduction test (FECRT); however this test has not been validated for *P. equorum* yet. Moreover, this test provides no information regarding the proportion of resistant individuals in the population and has a high risk to be false negative when the frequency of resistant parasites is still low [30]. Additionally, results are only available after two weeks.

In order to find predictive molecular markers, parasites have to be screened for genetic changes. As IVM is a known substrate of mammalian Pgps [31], the objective of this work was to identify and characterize *P. equorum* Pgps. Remarkably, SNPs in non-drug target genes potentially involved in the development of ML resistance were identified by comparing the coding sequences for specific Pgps between several populations with different IVM susceptibility. Furthermore, increased Pgp expression levels were encountered in a *P. equorum* isolate showing reduced IVM-susceptibility.

## Materials and Methods

### Parasite Material

Adult *P. equorum* worms were obtained from a necropsy of experimentally infected horses which were euthanized by bleeding through opening the carotid artery immediately following captive bolt stunning. Worms were stored at −80°C until use. Experiments were conducted in accordance with the German law (Tierschutzgesetz) and the European Union directive 2010/63/EU regarding animal welfare and approved by the Landesamt für Verbraucherschutz und Lebensmittelsicherheit (LAVES) under the reference number 33.9-42502-05-07A499. Fecal samples were collected rectally from horses of six stud farms and were sent in the institute for routine diagnostic examinations. The remainders of the samples not used for diagnostic purposes were used for ascarid egg collection and thus no permit for sample collection was required according to current legislation. Two populations (collected from stud-farm A and B-1) were susceptible against ML and treatment with IVM resulted in complete elimination of *P. equorum* eggs in feces two weeks after treatment. From stud farm A eggs were collected in two sequential years showing no difference in ML susceptibility *in vivo*. For foals of stud farm B, no reduction but even an increase of the fecal egg count was found one year later when routine diagnostic controls after deworming with IVM were carried out by Flotac for 33% of the foals (n = 9). For this reason, eggs from the same stud farm but collected at different points of time were classified as two populations in further investigations (B-1 and B-2). Besides eggs from farm B-2, the *P. equorum* population on an additional farm (E) did show decreased IVM susceptibility at the FECRT (i.e. FECR 0%; n = 9). Samples of this farm were also taken in two different years with no hints of changes in ML susceptibility *in vivo*. Two other stud-farms (C and D) were not definitely classified being either IVM susceptible or having a decreased susceptibility as they just showed initial signs of decreased ML susceptibility: Repeated treatment with MOX was not successful for 17% of the foals (n = 23) of stud farm D showing a FECR of 89.6% as determined by Flotac 19 days after treatment. At stud farm C, 22% of the examined foals (n = 23) were still highly positive for *P. equorum* in the fecal examination 20 days after deworming with IVM (52–200 eggs per gram). Unfortunately, no pre-treatment data were available for this farm, so determination of the FECR was not possible. Ages of the examined foals ranged from four to eight months. Eggs were isolated from feces by sieving with different mesh sizes (120 µm and 80 µm, respectively) followed by sedimentation and flotation with saturated saline before eggs were diluted with tap water and stored in ventilated cell culture bottles at 4°C.

For quantitative reverse transcriptase-PCR, adult and pre-adult worms with unknown treatment of the infected horses were collected from a slaughterhouse in Slomniki near Kraków, Poland with permission by the slaughterhouse manager to isolate and use the ascarids for further analyses. A group of pre-adult worms which were suspected to be ML resistant was obtained from Prof. Bretislav Koudela (University of Veterinary and Pharmaceutical Sciences Brno, Czech Republic). These worms were collected from the small intestine of a three month old foal kept on a farm where horses were regularly treated with IVM and where ML resistance was suspected due to the persistence of egg shedding post treatment (though no FECRT available). Pre-adult worms had a mean length of approximately 10 cm which suggests that the animal was infected within the first days after birth [32]. This group was compared with a randomly selected, size-matched group of worms from the slaughterhouse in Kraków.

### Cloning and Sequencing of *Peq*Pgps

Total RNA was extracted from adult female worms using peqGOLD TriFast™ (Peqlab), following the manufacturer’s instructions, after homogenization with a TissueRuptor (Qiagen). RNA was stored at −80°C until use.

Contaminating genomic DNA was eliminated by digestion with DNase I (Fermentas). First strand cDNA synthesis was performed with 160 ng of total RNA using the RevertAid™ Premium Reverse Transcriptase Kit (Fermentas) and random hexamer primers according to the manufacturer’s protocol with incubation at 22°C for 5 min followed by 50°C for 30 min, 60°C for 30 min and 85°C for 5 min. A nested PCR with degenerated primers (Table S1) [18] was performed to generate an initial PCR product of the internucleotide binding domain (IBD). The reaction contained 2.5 µl AccuPrime™ Buffer I (with dNTPs), 1 µM of each forward and reverse primers, 0.5 µl cDNA, 0.5 µl AccuPrime™ Taq DNA Polymerase (Invitrogen) and 16.5 µl dH_2_O. After an initial denaturation at 94°C for 2 min, 40 cycles with 94°C for 15 s, 55°C for 30 s and 72°C for 1 min were performed. Gel-purified PCR products were cloned into the pCR4 TOPO vector (Invitrogen). Sequencing was carried out by GATC Biotech (Konstanz). Gene specific primers (Table S1) were designed from the sequenced fragments for RACE PCR (5'/3' RACE Kit, 2^nd^ Generation, ROCHE), which allowed amplification of the respective full-length cDNA sequences.

### Phylogenetic Analyses

For phylogenetic analysis, nucleic acid sequences were translated into amino acid sequences and these were compared with Pgp amino acid sequences of other nematodes as well as sequences of *Mus musculus*, *Schistosoma mansoni*, *Pediculus humanus corporis*, *Drosophila melanogaster*, *Mytilus galloprovincialis* and *Mytilus californianus* as outgroup (for Genbank-accession numbers see Table S5). Sequences were first aligned with ClustalX2 using default parameters. The alignment was then analyzed with Prottest 3.0 [33], [34] to identify the optimal amino acid substitution model. The number of substitution rate categories for this analysis was set to 8. Finally, a maximum likelihood tree was calculated using PhyML 3.0.1 [35], [36] with 8 substitution rate categories and the LG+G+F model [37]. Both, nearest neighbor interchange (NNI) and subtree pruning and regraftment (SPR) moves were allowed. Calculations were started with one neighbor joining and five random trees to avoid trapping of the iterative optimization process in a local maximum of the likelihood function. Both the Shimodaira-Hasegawa [SH] approximate likelihood test and the Baysian transformation of the approximate likelihood ratio test for branch support were calculated. The best tree was finally visualized using MEGA5.

### SeqDoC Analyses

The six *P. equorum* populations with different ML susceptibility were compared regarding their Pgp cDNA sequences. For each population at least to independently obtained samples were analyzed. For RNA isolation approximately 5000 *P. equorum* eggs were crushed in tubes containing Buffer RL1 (Macherey & Nagel) and ceramic beads of 0.4 to 0.6 mm (innuSpeed Lysis Tube S, Analytik Jena, Germany) with a Speed Mill homogenizer (Analytik Jena). Afterwards, RNA was isolated using the NucleoSpin RNA XS-Kit (Macherey & Nagel) including a DNase treatment step during isolation. Additionally, RNA of individual pre-adult putatively resistant (Czech Republic worms) and randomly selected worms (from slaughterhouse in Krakow) was extracted with TriFast™ (Peqlab). RNA was reverse transcribed into cDNA with RevertAid™ Premium First Strand cDNA Synthesis Kit (Thermo Scientific) according to the manufacturer’s protocol. RT-PCR was performed in a 10 µl reaction (1 µl AccuPrime™ Buffer I, 0.5 µM forward and reverse primers, 1 µl cDNA, 0.25 µl AccuPrime™ Taq DNA polymerase (Invitrogen) and 4.75 µl dH_2_O. For each Pgp sequence, eight pairs of gene-specific primer pairs covering the whole open reading frame with about 100 bp overlap between amplicons were used (Table S2). Cycling conditions were first an initial denaturation at 94°C for 2 min, then 40 cycles with 94°C for 15 s, 55°C for 30 s and 72°C for 45 s. PCR fragments of about 700 bp were excised from agarose gels, purified and directly sequenced using the gene-specific forward primer. For each of the eight overlapping fragments, sequence chromatogram files were entered into the SeqDoC web platform [38]. The detected SNPs were mapped to the full-length cDNA sequence and translated to discriminate silent and missense mutations and to find any potential amino acid changes. The chemical distance between these substitutions was determined according to Grantham [39]. Additionally, chromatogram files of sequences containing SNPs potentially involved in ML resistance were exported to GraphPad Prism 5.0.3 and area under curve of fluorescence signals detected for the corresponding bases was calculated.

### Three-dimensional Modelling of Pgp-11

The webserver ESyPred2d was used to calculate a tertiary structure model using a 3D model of murine Pgp-1 deposited in PDB with accession no. 3G5U_A [40] as template. The model obtained for *P. equorum* Pgp-11 was visualized using Jmol, an open-source Java viewer for chemical structures in 3D (http://www.jmol.org/).

### Real-time RT-PCR of *P. equorum* Eggs from Different Populations

For comparison of *P. equorum* Pgp expression levels in the different populations, quantitative RT-PCR was conducted. Embryonated *P. equorum* eggs of populations A-E were homogenized and RNA was subsequently extracted with the innuPREP RNA Mini Kit (Analytik Jena) which already includes a genomic DNA removal step. RNA purity and amount were measured with a Take3 plate in a Synergy plate reader (Biotek). Due to the very small amount of RNA isolated from eggs, control of RNA integrity on the Bioanalyzer® was not possible. For each population approximately 80 ng RNA were used for the following cDNA synthesis with QuantiTect Reverse Transcription Kit (Qiagen) per 20 µl reaction with another integrated genomic DNA removal step. Real-time RT-PCR was performed in a total reaction of 50 µl with 25 µl GoTaq qPCR Master Mix (Promega), 0.2 µM each of a forward and reverse gene-specific primer for the particular *P. equorum* Pgp (Table S3), 18 µl dH_2_0 and 5 µl of template on white 96-well plates sealed with BZO adhesive optical film (Biozym, Germany). Reactions were carried out under the following PCR cycling conditions: After an initial denaturation step at 95°C for 2 min, 50 cycles with 95°C for 15 s, 59°C for 30 s and 72°C for 30 s were performed on a CFX96 Thermal Cycler (Biorad). Melting curves were recorded between 60°C and 95°C. Two biological replicates (from different sampling occasions) were analyzed for each of the five populations; for each replicate two cDNA syntheses were performed and each sample was analyzed in duplicates on the same plate (technical replicates). At least four independent PCR runs were performed, providing a minimum of eight replicates. Three RNAs, namely actin, glyceraldehyde 3-phosphate dehydrogenase (gpd-1) and 18S rRNA, were used as reference genes for normalization according to MIQE guidelines [41]. Serial dilutions of plasmid DNA (from 4 to 4×10^6^ copies per reaction) were used to obtain standard curves and calculate reaction efficiencies. Pgp gene expression was normalized to the geometric mean of the signals obtained from the reference genes as described by Hellemans et al. [42].

### Real-time RT-PCR of Different *P. equorum* Tissues

A real-time RT-PCR was conducted to compare pgp-11 and pgp-16 mRNA expression between tissues. For this purpose, adult male and female worms were collected from the slaughterhouse (Kraków, Poland) and stored at −80°C in RNA*later* (Sigma). Worms were dissected and from a middle body part, uterus, intestine and body wall of six female worms and the intestine and body wall of six male worms were collected. Unfortunately, clean preparation of testes was not possible with the material preserved in RNA*later*. Homogenization of body wall and uterus was carried out in tubes containing ceramic beads of 1.4–1.6 mm, while the intestine was homogenized in tubes with ceramic beads with a size of 0.4–0.6 mm (Analytik Jena, Germany). RNA isolation, cDNA-synthesis and real-time RT-PCR were performed as described above. To ensure that no RNA degradation would influence results, RNA integrity was confirmed with the Bioanalyzer 2100 (Agilent Technologies). Only RNA preparations with a RNA integrity number (RIN) >8.3 were used.

### Real-time RT-PCR of Adult *P. equorum* Worms after Incubation with IVM

In order to investigate the influence of a short term incubation of *P. equorum* with IVM, adult worms were collected at an abattoir (Kraków, Poland), sexed and rinsed with pre-warmed artificial perienteric fluid (APF) to remove host intestinal contents as previously described [43]. In brief, APF contained 5 mM MgCl_2_, 6 mM CaCl_2_, 24 mM KCl, 23 mM NaCl, 110 mM NaCH_3_COO, 11 mM dextrose, 10 mM Tris and was adjusted to a final pH of about 7.5 at 37°C. Before IVM-challenge was carried out in ventilated cell culture flasks, worms were adapted to *in vitro* conditions at 37°C in APF for 18 h. Providing 20 ml of APF for each worm with either IVM (10^−8^ M or 10^−9^ M) or only 1% DMSO (vehicle control), worms were incubated for 12 h at 37°C. At a concentration of 10^−9^ M nearly all worms survived the treatment whereas higher IVM concentrations killed worms before the end of the incubation time. Therefore, only worms surviving exposure to 10^−9^ M IVM were used for quantitative analysis of gene expression, rapidly frozen and stored at −80°C until further use. Total RNA was extracted from individual female worms using the TriFast reagent as described above and RNA integrity was determined with the Bioanalyzer 2100 (RIN >9.1) before cDNA was synthesized. Further real-time RT-PCR steps were conducted as described above. Five biological replicates (individual worms) were analyzed per group and for each RNA preparation of individual worms, two cDNA syntheses were performed. PCR was carried out with two technical replicates resulting in at least four replicates per individual worm.

### Comparison of Expression Levels between a Putatively Resistant and Random Group of *P. equorum*

Worms of a presumably IVM resistant population were collected from the small intestine of a foal and directly frozen at −80°C. Samples of a group of pre-adult *P. equorum* worms with unknown treatment were collected from the slaughterhouse in Kraków, Poland. For gender determination, degenerated primers for vitellogenin-6 derived from corresponding sequences of *Ascaris suum*, *C. elegans* and *C. briggsae* (Table S4) were used in an RT-PCR to differentiate between female and male worms. Relative expression of pgp-11 and pgp-16 of each group containing five worms was compared with regard to the state of resistance of worms in a RT-qPCR. RNA quality was assessed and only RNAs with RIN >9.1 were used for downstream application. For each biological replicate, two separate cDNA synthesis were carried out, followed by two technical replicates for each cDNA.

## Results

### Sequencing of *P. equorum* Pgps

**Figure 1:**
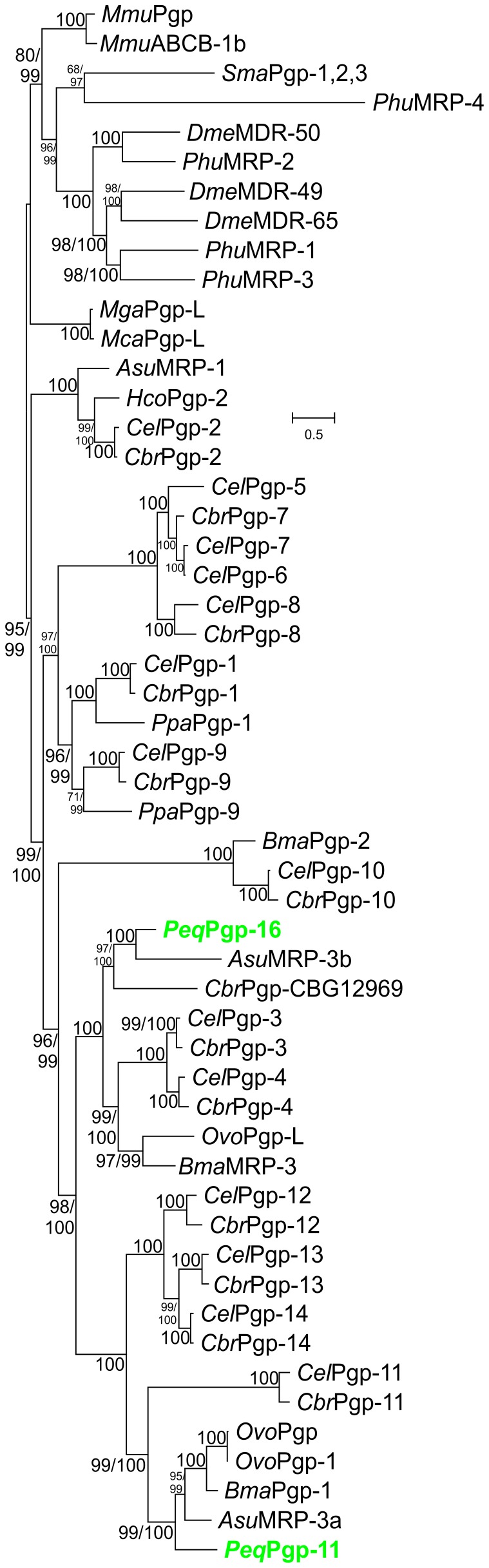
Maximum likelihood phylogenetic analysis of Pgp protein sequences from nematodes. Protein sequences were aligned using ClustalX2 under default conditions. Optimal amino acid substitution models were determined using Prottest 3.0 before calculating the most likely tree topology using PhyML3.0.1. The substitution model LG was used and PhyML was set to optimize the number of invariant sites, the amino acid frequencies and the Γ shape parameter for modelling the distribution of the amino acid substitution rate categories which were set to eight. Both results of the approximate likelihood ratio test modified according to Shimodaira and Hasegawa (before the slash) and of a Bayesian-like transformation of the approximate likelihood ratio test (after the slash) are shown as branch supports. As outgroup, Pgp sequences from mouse (*Mmu*), *Drosophila melanogaster* (*Dme*), *Pediculus humanus corporis* (*Phu*), Schistosoma mansoni (*Sma*) were used. The complete Pgp protein families of *Caenorhabditis elegans* (*Cel*) and *Caenorhabditis briggsae* (*Cbr*) were included. In addition, available annotated Pgp sequences of *Pristionchus pacificus* (*Ppa*) and of the parasites *Ascaris suum* (*Asu*), *Brugia malayi* (*Bma*), *Haemonchus contortus* (*Hco*) and *Onchocerca volvolus* (*Ovo*) were used for alignment. Pgp, P-glycoprotein; MDR, multi-drug resistance protein. The scale bar represents the indicated number of substitutions per site. Accession numbers for protein sequences in the tree are provided in Table S5.

RT-PCR using degenerated primers resulted in 12 fragments localized in the IBDs of Pgps. These products had a size of approximately 408 bp. Identity of the obtained PCR products was confirmed by a nested PCR resulting in products of about 345 bp. Following 5'/3' RACE starting from two selected partial sequences allowed obtaining full-length sequences with a length of 3858 bp and 3855 bp, respectively. For the first sequence, a 5' spliced leader sequence SL1 [44] was identified upstream of the open reading frame. Sequences were deposited in the GenBank™ database with accession no. JX308230 and JX308231. Conceptional translation resulted in two putative protein products of 1285 and 1284 amino acids revealing typical, highly conserved Pgp domains such as two Walker A/P-loops, Walker B motif, Q-loop/lids, D-loop, H-loops/switch region, ABC transporter signature motifs as well as two ATP binding sites (Fig. S1). Predicted proteins have a calculated molecular weight of 141.05 kD and 139.45 kD, respectively. Phylogenetic analysis by maximum likelihood estimation using the deduced amino acid sequences from the two putative Pgps of *P. equorum* and Pgp sequences of several nematodes including the complete Pgp sets of *C. elegans* and *Caenorhabditis briggsae* (Table S5) revealed that nematode Pgps are monophyletic and did not evolve from different precursors present in a common protostomian ancestor. The first *P. equorum* Pgp sequence is orthologous to *C. elegans* Pgp-11 (accession-no.: NP_495674) (Fig. 1) and was therefore designated as *Peq*Pgp-11. Blast analyses showed an identity of 37%. The other full-length sequence has no direct ortholog in the *C. elegans* Pgp-family, but is closely related to the *Caenorhabditis briggsae* CBG12969 sequence (accession-no.: XP_002630530). In the *C. elegans* genome, Pgp-3 and Pgp-4 (accession-no.:NP_509901 and NP_001257143) with an identity of 51% and 49%, respectively, were most closely related. According to the rules for gene nomenclature in nematodes [45] it was designated as *Peq*Pgp-16. The most closely related sequences identified in GenBank were the *A. suum* Pgp orthologs MRP-1 (accession-no.: ADY40818) and MRP-3 (accession-no.:ADY40776) with 92% identity (99% similarity) and 93% identity (91% similarity) to *Peq*Pgp-11 and *Peq*Pgp-16, respectively.

### Single Nucleotide Polymorphisms in *P. equorum* Pgps

In order to compare the cDNA sequence of the two Pgps between different *P. equorum* populations, gene specific primer sets were used to amplify overlapping fragments of approximately 700 bp covering the complete coding sequences followed by sequencing and SeqDoC analysis. RNA was isolated from eggs collected from two sensitive (farms A and B1), two intermediate (farms C and D) and two populations with severely decreased susceptibility (farms B2 and E) to MLs. Several single nucleotide polymorphisms (SNP) could be identified in this way.

For *Peq*pgp-16 cDNA, the analysis revealed only seven SNPs (Table S7 and Fig. S1), two of them missense and five silent mutations, but none of them was consistently present in all investigated populations with decreased ML susceptibility.

In contrast, the corresponding analysis of the *Peq*pgp-11 cDNA revealed 29 polymorphisms by comparing the partial sequences of all populations (Table S6 and Fig. S1). Fifteen SNPs resulted in a silent mutation, whereas 14 caused amino acid substitutions. Most of them were localized in the NH_2_-terminal part of the first transmembrane domain region whereas three of these missense SNPs were found in the COOH-terminal part of the second transmembrane domain region within a range of about 50 amino acids (Fig. S1). In IVM susceptible and intermediate populations both alleles were identified at all three positions although the frequency of the minor allele in the susceptible population was strongly increased in the intermediate populations (Fig. S2 and Table 1). In contrast, populations with decreased susceptibility were homozygous and revealed exclusively that allele which was present at only about 20–25% in the susceptible populations. All three SNPs caused a change in the deduced amino acid sequence, *i.e.* from aspartic acid to asparagine (Asp931Asn), from cysteine to serine (Cys951Ser) and from tyrosine to histidine (Tyr978His). The chemical differences according to the scale of Grantham [39] between these amino acids are 23, 112 and 83, respectively (Table S6). The first and the last polymorphism were caused by a substitution at the first position of the codon, while the substitution of the second amino acid was a result of a change in the second codon position. A three dimensional model of *Peq*Pgp-11 was calculated using the ESyPred3D webserver [46] and mouse Pgp-1 (PDB accession no.: 3G5U_A) as template. Fig. S3A reflects the position of the three substituted amino acids and highlights their proximity to residues known to be involved in drug binding in the mouse Pgp. The latter were described to interact with the Pgp substrates QZ59-SSS, QZ59-RRR or verapamil [40] and the corresponding amino acids in *Peq*Pgp-11 were identified from a local alignment of both sequences (Fig. S3B). Interestingly, only nine out of 27 residues involved in substrate binding are conserved between both proteins suggesting that there are substantial differences in the properties of the substrate binding pockets of both transporters.

**Table 1:** Allele frequencies in different *Parascaris equorum* populations.

		Population^a^							
Position	N^b^	A	B1	B2	C	D	E	Czech^c^	Poland^d^
2791	GA	7822	8020	−100	62.737.3	4456	−100	58.741.3	72.827.2
2852	GC	79.320.7	75.824.2	−100	61.338.7	42.757.3	−100	56.143.9	75.324.7
2932	TC	71.628.4	63.236.8	−100	5050	32.367.7	−100	45.954.1	60.339.7

a Populations from farms are encoded only by letter code used throughout the manuscript. Values were obtained from the area under the curve of the corresponding pgp-11 sequence chromatograms of *P. equorum* populations showing different ML susceptibility. All values are given in %.

b Nucleotide.

c Putatively resistant pre-adult worms from Czech Republic.

d Randomly selected pre-adult worms from Krakow.

### Tissue-specific Expression Patterns of pgp mRNAs

**Figure 2:**
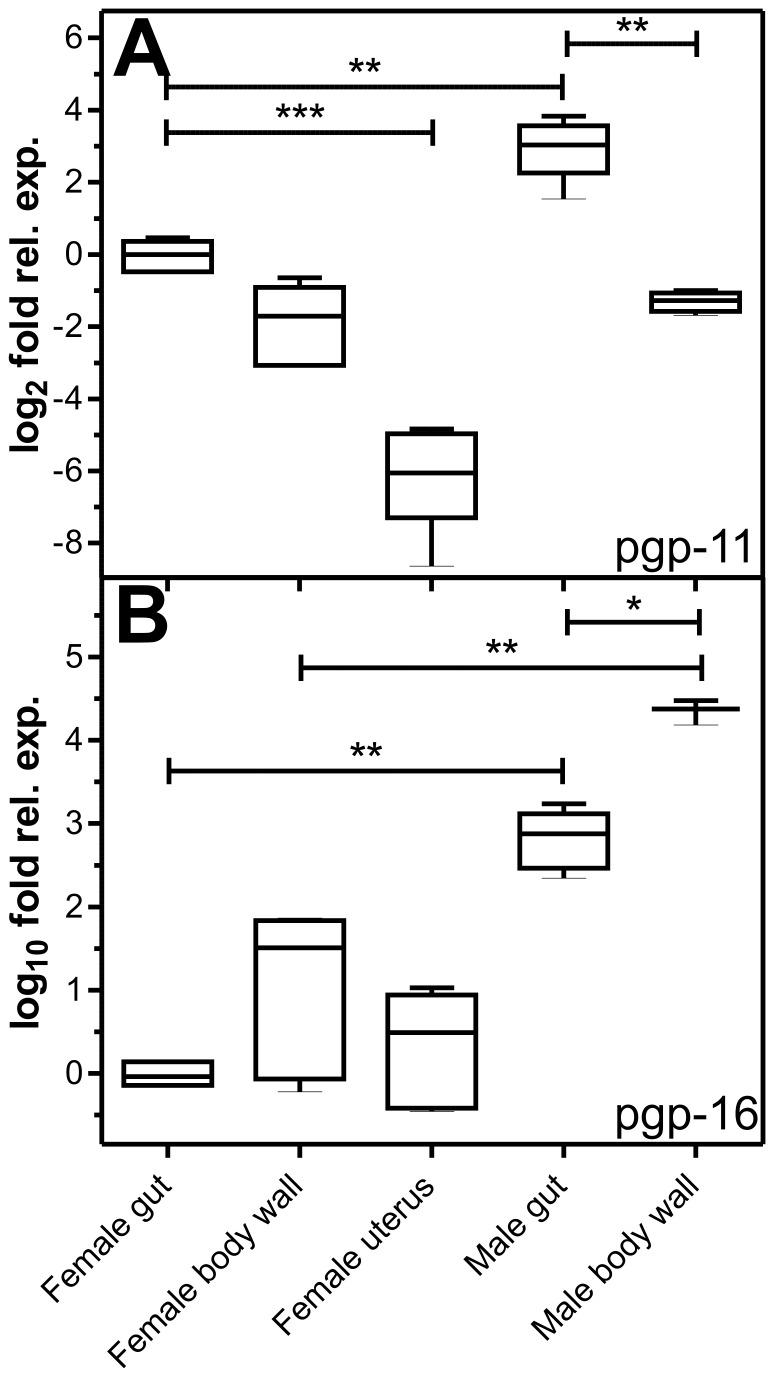
Tissue expression pattern of *P.* *equorum* pgp mRNAs. Expression of pgp-11 (A) and pgp-16 (B) mRNAs was measured in gut, body wall, and uterus of 5–6 female and male worms. Real-time RT-PCR was performed on individual worms using actin and gpd-1 mRNA and 18S rRNA as reference genes. Two biological replicates were investigated for each population and each replicate was analyzed in two technical repeats of two independent cDNA samples. All individual values were normalised to the mean of the respective controls. Whiskers represent minimal and maximal values. A Kruskal-Wallis test with Dunn’s post hoc test was performed to identify significant differences between the three female tissues. Mann-Whitney U tests were conducted to identify differences between the male tissues and between males and females for the same tissue. ***, p<0.001; **, p<0.01; *, p<0.05.

Comparison of expression levels for both pgp mRNAs identified several significant differences. In general, differences in expression levels were much higher for pgp-16 than for pgp-11 (Fig. 2). For pgp-11, significantly lower expression was found in the uterus compared to the female gut, and for the male body wall compared to the male gut (Fig. 2A). Moreover, expression was higher in male than female gut. The pgp-16 mRNA expression was higher in male than in corresponding female tissues and in male body wall than in male gut (Fig. 2B).

### Expression Levels of pgp mRNAs in Eggs of Different *P. equorum* Populations

**Figure 3:**
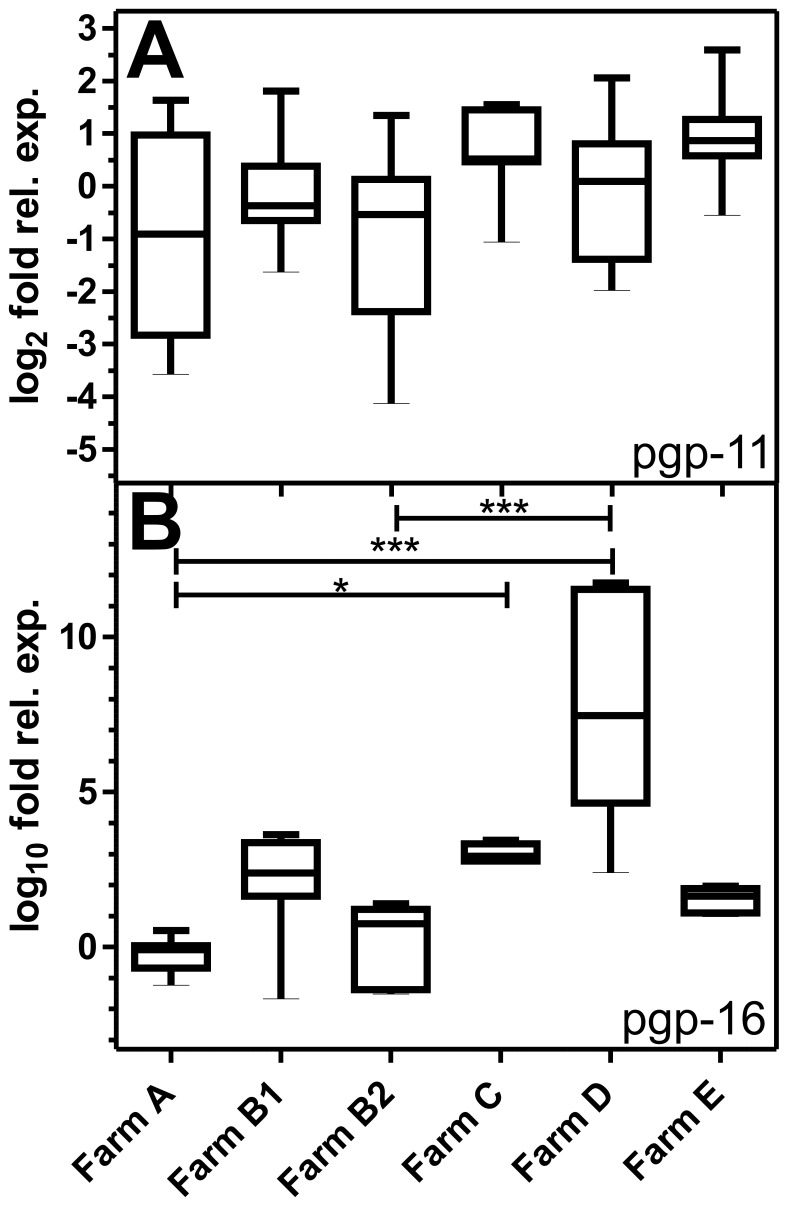
Expression of pgp-11 (A) and pgp-16 (B) mRNAs in *Parascaris equorum* eggs. Expression of mRNAs was compared between populations showing full (farms A and B1), intermediate (farms C and D) or decreased (farms B2 and E) ML susceptibility. RNA was extracted from eggs obtained from five different stables (farms A-E). Farm B was represented twice, once when IVM was still fully effective (farm B1) and a second time when efficacy was severely compromised (farm B2). Real-time RT-PCR was performed in four biological replicates and two technical replicates. Actin and gpd-1 mRNA and 18S rRNA were used as reference genes. All individual values were normalized to the mean of the respective controls. Whiskers represent minimal and maximal values. A Kruskal-Wallis test with Dunn’s post hoc test was performed to identify significant differences between the six farms. ***, p<0.001; *, p<0.05.

Constitutive expression-levels of both Pgps in eggs from the six populations with different ML susceptibility were measured by RT-qPCR. All expression levels were normalized to the susceptible *P. equorum* population from stud-farm A. Variation between the biological replicates was very small whereas huge differences in the expression levels of the pgp-11 and pgp-16 transcripts were observed between the different populations (Fig. 3). However, no consistent patterns of Pgp-expression between ML susceptible populations on one hand and populations with decreased IVM susceptibility on the other hand were observed.

### *In vitro* Effects of IVM on pgp mRNA Expression in Adult Worms

**Figure 4:**
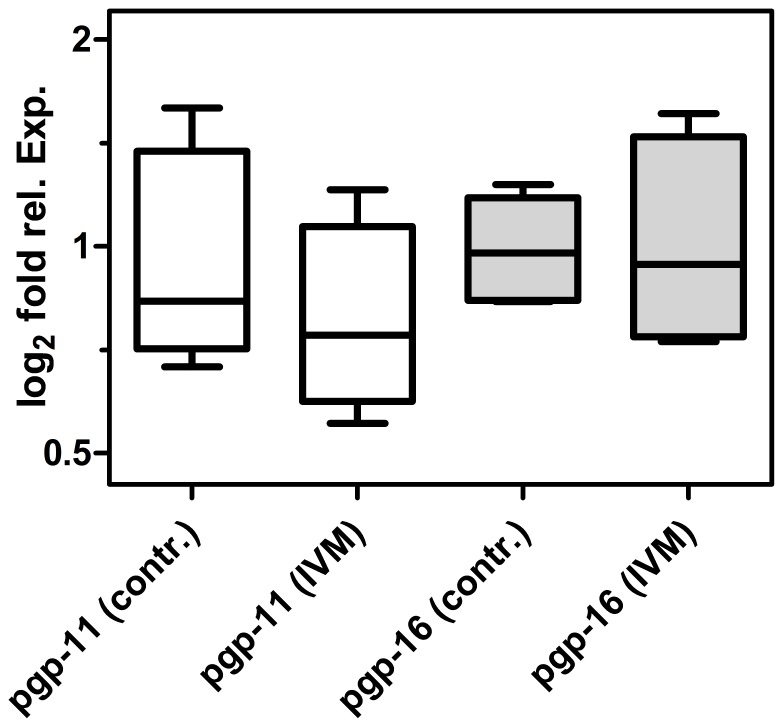
Effects of *in vitro* IVM incubation on Pgp mRNA expression. Adult male *P. equorum* (n = 5) were incubated for 18 h in 10^−8^ M IVM (IVM) or only in 1% DMSO as vehicle (contr.). Real-time RT-PCR was performed on individual worms using actin and gpd-1 mRNA and 18S rRNA as reference genes. Expression of pgp-11 (open boxes) and pgp-16 (grey boxes) are shown. At least two cDNA syntheses were made for each biological replicate and used for two technical replicates each. All individual values were normalized to the mean of the respective controls. Whiskers represent minimal and maximal values. No significant differences were found.

Adult *P. equorum* from a phenotypically uncharacterized Polish field population were cultured *in vitro* in the presence of 10^−8^ and 10^−9^ M IVM for 18 h before RNA isolation. All worms incubated in 10^−8^ M IVM died during incubation. In contrast, most worms incubated in 10^−9^ M IVM survived and RNA was extracted only from worms that were still motile after 18 h to prevent that potential degradation of RNA could compromise the analyses. Real-time RT-PCR did not show any significant differences between controls and IVM exposed individuals regarding pgp-11 and pgp-16 mRNAs (Fig. 4).

### Comparison of a Putatively Resistant and a Random Group of *P. equorum*

Two groups of randomly selected male and female worms of young adult *P. equorum* were compared to a group of worms of the same development stage but with an assumed resistant phenotype due to treatment failure. Sequence comparison using the SeqDoC method revealed for the random group a genotype corresponding to the one of the susceptible *P. equorum* populations described above. In contrast, the group showing the resistant phenotype revealed frequencies of the three SNP correlating with decreased susceptibility between 41.3% and 54.1% (Table 1).

**Figure 5:**
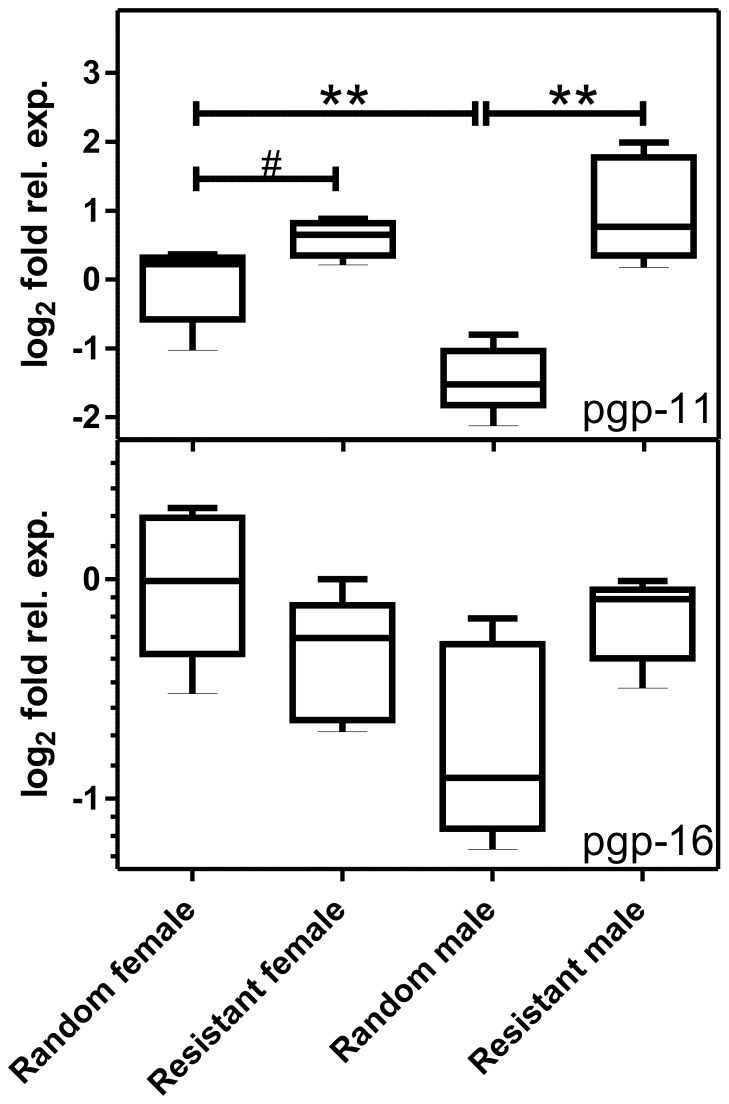
Expression of pgp-11 and pgp-16 mRNAs in pre-adult *Parascaris equorum*. A randomly chosen group (n = 5) was compared with a group where ML treatment failed (n = 5). As a reference, expression of the actin, gpd-1 and 18S rRNA genes was measured. Real-time RT-PCR was conducted in duplicate for each sample using two different cDNAs for each biological replicate. Differences between randomly selected and putatively resistant worms were identified with Mann-Whitney U tests. **, p<0.01; *, p<0.05; #, p<0.056.

Resistance was associated with a significant 5.9 fold increase of pgp-11 expression in male worms (p = 0.008) while a 1.5 fold higher expression in putatively resistant female worms was just not significant (p = 0.056) (Fig. 5A). Moreover, a significant difference was found by comparing male and female worms within the random groups showing an 2.7 fold higher expression of Pgp-11 in female than in male worms (p-value <0.016), but not by comparing both genders between putatively resistant worms. The pgp-16 mRNA expression did not show any significant differences associated with resistance of worms (Fig. 5B).

## Discussion

Anthelmintic resistance in nematodes is an increasing problem in livestock but is also seen or is suspected to occur in human parasites such as *O. volvulus* [4], [47] and *Trichuris trichiura* [48]. During the past years, once very powerful broad-spectrum-anthelmintics such as IVM had to forfeit their efficacy against several parasite species. Therefore, it is important to characterize the mechanisms which are responsible for this development in order to (I) prolong the activity of existing drugs and (II) be aware of these mechanisms for the development of novel, resistance-breaking drugs in the future. ML resistance in nematodes has often been proposed to be associated with Pgp-mediated drug efflux [3] and for several organisms, these proteins and their sequences were already partially characterized. However, this was not yet the case for *P. equorum*, although this nematode showed a decreased susceptibility against MLs in several studies [23]. It is noteworthy that for *P. equorum,* like for most other parasitic nematodes, no laboratory animal or *in vitro* life cycle model is available to date. This is a major constraint for the study of mechanistic or functional processes in this parasite. Furthermore, such work is hampered by the very high costs and ethical limitations when using horses in experimental studies. Accordingly also in the present study it was only possible to use parasite material derived from field infections and consequently differing genetic background as well as only comparatively limited opportunities for phenotypic characterization.

The two Pgp cDNAs identified in this work provide the first information about *P. equorum* multi-drug transporters. It does not surprise that phylogenetic analyses revealed for both sequences a close resemblance to amino acid sequences of Pgps from the phylogenetically most closely related *A. suum*. However, for these transporters no further information except for the sequences is available. Comparison with corresponding sequences of other nematodes showed also a close relationship of *Peq*Pgp-11 to *C. elegans* Pgp-11 as well as to *O. volvulus* Pgp and Pgp-1 which have already been linked to ML resistance [21], [49]. In order to prevent any confusion, it should probably be mentioned that both *O. volvolus* cDNAs were not designated according to orthology with *C. elegans* orthologs. *C. elegans* Pgp-11 was found to be expressed in the excretory cell and in the intestine [50]. Similar locations were identified for the ortholog of *P. equorum* where the examination of different tissues for their Pgp expression levels revealed that in both genders a significantly higher expression was found in the intestine compared to body wall and uterus. The sequence of *Peq*Pgp-16 is apparently an ortholog to *C. briggsae* CBG12969 and is relatively closely related to *C. elegans* and *C. briggsae* Pgp-4 and Pgp-3. The *C. elegans pgp-3* gene has been shown to be involved in protection against toxic compounds such as colchicine and chloroquine [7] as well as against bacterial toxins [51]. More recently, an association between overexpression of *C. elegans pgp-4* and IVM resistance was demonstrated [14]. For *C. elegans pgp-4*, the excretory cell was identified as the main expression site, whereas results for *C. elegans pgp-3* indicate expression predominantly in the intestine and the excretory cell [50]. Results for *P. equorum pgp-16*, revealed a high expression in the body wall containing not only muscle cells and hypodermis but also the excretory cell. Further *in situ* hybridization analyses will be required to identify the cellular source of pgp-16 mRNA in the body wall.

Pgps might contribute to the development of resistance by either overexpression [10], [20], [52] or changes in their primary structure that could lead to accelerated transport of anthelmintics. Comparison of two ML susceptible, two intermediate and two populations with decreased ML susceptibility revealed only a few mutations for *Peq*Pgp-16, most of them silent. Regarding pgp-16 expression levels, no consistent up- or down-regulation of pgp-16 correlating with decreased ML susceptibility was found. Furthermore, in another experiment using a phenotypically uncharacterized field population IVM exposure *in vitro* did also not increase its expression. We therefore conclude that PeqPgp-16 does not contribute to decreased ML susceptibility at least in the populations studied here.

In contrast, corresponding studies on Pgp-11 imply a putative contribution to ML resistance of this transporter. In particular, besides several other coding and silent SNPs, three amino acid substitutions were strongly increased in frequency or exclusively present in populations with intermediate and decreased ML susceptibility, respectively.

Aller et al. [40] described the three-dimensional structure of the mouse Pgp (ABCB1) with drug-binding sites in the internal cavity. Mapping of these amino acid residues and the three resistance-correlated exchanges to a calculated 3D model of *P. equorum* Pgp-11 revealed that the three amino acid residues are close to or in the putative drug binding region. Increased IVM affinity of Pgp-11 due to these mutations is an attractive hypothesis that could explain why such SNPs have apparently been selected in IVM resistant populations. Further experimental work involving recombinant *P. equorum* Pgp-11 from susceptible and resistant populations and characterization of their transport properties will be required to corroborate this hypothesis. There are currently no guidelines from the World’s Association for Advancement of Veterinary Parasitology (WAAVP) for conducting the FECRT in *P. equorum*, in part due to the facts that there is (I) no correlation between worm burdens and egg counts [53] and (II) a high variability in egg counts even in samples repeatedly taken from the same animal [32]. Nevertheless, FECRT data are currently considered to be the only way to at least roughly estimate resistance. In particular since there is no standardized protocol for detection of resistance against ML, the SNPs and expression differences identified here can well be the basis for development of molecular tests as previously described for resistance to benzimidazoles [54]. In the absence of any validated *in vivo* or *in vitro* tests available for detection of resistance in ascarids, the opportunity to develop and evaluate molecular tests using the data provided here might be important also for human diseases such as ascarosis and toxascarosis. Validating and using the identified SNPs as molecular markers for resistance using for instance comparison of systematic comparison of FECRT data and SNP frequencies (*e.g.* by pyrosequencing) could solve this problem.

Increased expression of *Peq*pgp-11 would further substantiate the view that Pgp-11 contributes to IVM resistance in *P. equorum*. However, incubation of worms *in vitro* for 12 h did not modify pgp-11 mRNA expression. Moreover, *P. equorum* eggs were investigated by real-time RT-PCR, but no correlation between ML tolerance and pgp-11 mRNA levels was found. In marked contrast, pre-adult worms obtained from a presumably resistant *P. equorum* population showed significant overexpression of pgp-11 mRNA in male and female worms. In addition, the three SNPs identified in the eggs of other populations with decreased susceptibility were found with a frequency similar to that found in the intermediately resistant *P. equorum* populations. These findings imply that the susceptibility to MLs in *P. equorum* can be dependent on both changes in Pgp-11 primary structure and pgp-11 expression levels. Further studies involving more adult stages will reveal whether this gene generally shows higher expression levels in ML resistant worms.

The reason for the different Pgp-11 expression patterns of the two development stages might be explained by lower expression levels of Pgps in earlier development stages than in adults due to other protective mechanisms. The high tenacity of embryonated ascarid eggs is probably based on the eggshell constituting an effective barrier for most compounds. Therefore, exposure to xenobiotics and need for efflux effectors such as Pgps might be lower compared to free larvae and adult worms which are in direct contact to exogenous compounds. Comparison of non-feeding, relatively resistant third larvae of *H. contortus* showed similar results, *i.e.* no significant differences were found in expression of nine Pgp mRNAs between a resistant and susceptible strain [16]. These authors suggested, among noting that only constitutive mRNA levels were under investigation, expression may change throughout the life cycle. They conclude that developmental stages living in compartments with the highest risk for an IVM exposure require more protection through Pgps than stages without regular exposure to the drug. Indeed adult worms of *O. volvulus* were shown to have higher expression of two Pgp-genes than third stage larvae [21]. Nevertheless it should be mentioned that for other ML resistant nematodes such as *T. circumcincta* significant increases of the Pgp transcript levels were found throughout the whole life-cycle especially in the eggs [20]. Kerboeuf et al. [55] described significantly higher Pgp amounts on the egg-surface of *H. contortus* resistant to BZs and IVMs. These results are in apparent opposition to those reported in this study but the differences might be explained by different life cycles and differences in the egg structure between trichostrongylid and ascarid nematodes. Probably, there are different possible mechanisms to acquire ML resistance and various species (or even populations) might therefore differ in their actual resistance mechanisms.

In conclusion, this study suggests involvement of Pgp-11 in the level of IVM susceptibility in *P. equorum*. Increase in pgp-11 mRNA expression was observed in one putatively resistant population. Most importantly increased frequency of *pgp*-*11* alleles differing in three SNPs from the predominant alleles in susceptible populations was observed in several populations with decreased IVM susceptibility. Bourginat et al. [56], [57] identified SNPs in *Dirofilaria immitis* Pgps which are correlated to ML susceptibility/resistance. While one SNP was non-coding, the other caused a conservative change from lysine to arginine, immediately before the second nucleotide binding domain. Herein, we describe for the first time putatively ML resistance associated non-synonymous SNPs located in substrate binding regions of Pgps. This further corroborates the hypothesis that MLs select for certain Pgp genotypes. However, further functional analysis involving recombinant expression of both alleles and quantification of ML transport are required to prove involvement of the herein described SNPs in ML resistance in *P. equorum*. To date, molecular markers for detection of IVM resistance are urgently required and this study provides promising candidates obtained by comparison of field populations. Whether the identified SNPs might be useful as molecular markers for early detection of resistance will be evaluated by comparing additional field populations differing in geographical origin and resistance status. Moreover, analysis of corresponding regions in *pgp-11* genes from ML resistant nematodes of other species will further help to elucidate the involvement of Pgps in development of IVM resistance in general.

## Supporting Information

Figure S1 **Organization of the conserved domains and identified SNPs in** ***Peq*** **Pgp-11 and** ***Peq*** **Pgp-16.** ATP-binding sites are indicated in green, transmembrane domains in blue and further typical, conserved Pgp motifs in red. Length of the cDNA sequence is shaded in light grey. TMD, transmembrane domain; WA, Walker A/P-loop; WB, Walker B domain; WC, Walker C/linker peptide; D, D-loop; H, H-loop/switch region; Q, Q-loop/lid;
(PDF)

Figure S2 **Frequencies of alleles correlating with IVM susceptibility.** Chromatograms showing partial coding sequences of *Peq*Pgp-11 with positions of ML resistance associated SNPs of three farms of different ML susceptibilities: ML susceptible (farm A), intermediate ML susceptibility (farm C) and decreased ML susceptibility (farm E). Compared to the susceptible farms, farms of intermediate susceptibility had a tendency to the resistant genotype but still both alleles were present at all positions. In contrast, bases were replaced completely in farms of decreased susceptibility.
(PDF)

Figure S3 **Three dimensional model of** ***Peq*** **Pgp-11 structure.** (A) Residues corresponding to drug binding residues in the mouse Pgp-1 [40] (blue) and residues differing in PeqPgp-11 due to the three SNPs correlating with decreased IVM susceptibility (red) are highlighted in the 3D model. (B) Partial sequence alignments of mouse Pgp-1 and *Peq*Pgp-11 for regions involved in substrate binding. Identical and similar residues which are highlighted in dark grey and light grey, respectively. Amino acids known to be involved in substrate binding in *M. musculus* Pgp-1 are marked by asterisks.
(PDF)

Table S1 **Primer sets used for RACE PCR and amplification of two full length sequences of** ***P. equorum*** **Pgps.**
(DOCX)

Table S2 **Primer sets used for amplification of PCR products compared by SeqDoC analysis.**
(DOCX)

Table S3 **Primer pairs used for real-time PCR of** ***Peq*** **Pgp-11,** ***Peq*** **Pgp-16 and reference genes actin, gpd-1 and 18SrRNA.**
(DOCX)

Table S4 **Primer set for gender determination. Degenerated primers were generated from corresponding sequences of** ***Ascaris suum*** **,** ***C. elegans*** **and** ***C. briggsae*** **.**
(DOCX)

Table S5 **NCBI accession numbers and Worm Base ID for protein sequences used for maximum likelihood tree.**
(DOCX)

Table S6 **Position of single-nucleotide-polymorphisms in** ***Peq*** **Pgp-11 detected by SeqDoC analysis.**
(DOCX)

Table S7 **Position of single-nucleotide-polymorphisms in** ***Peq*** **Pgp-16 detected by SeqDoC analysis.**
(DOCX)
