# Supplementary figures and images for: Genetic Variants and Increased Expression of Parascaris equorum P-glycoprotein-11 in Populations with Decreased Ivermectin Susceptibility
Source: PLoS One. 2013 Apr 24;8(4):e61635. doi: 10.1371/journal.pone.0061635 (PMC3634834; doi:10.1371/journal.pone.0061635)

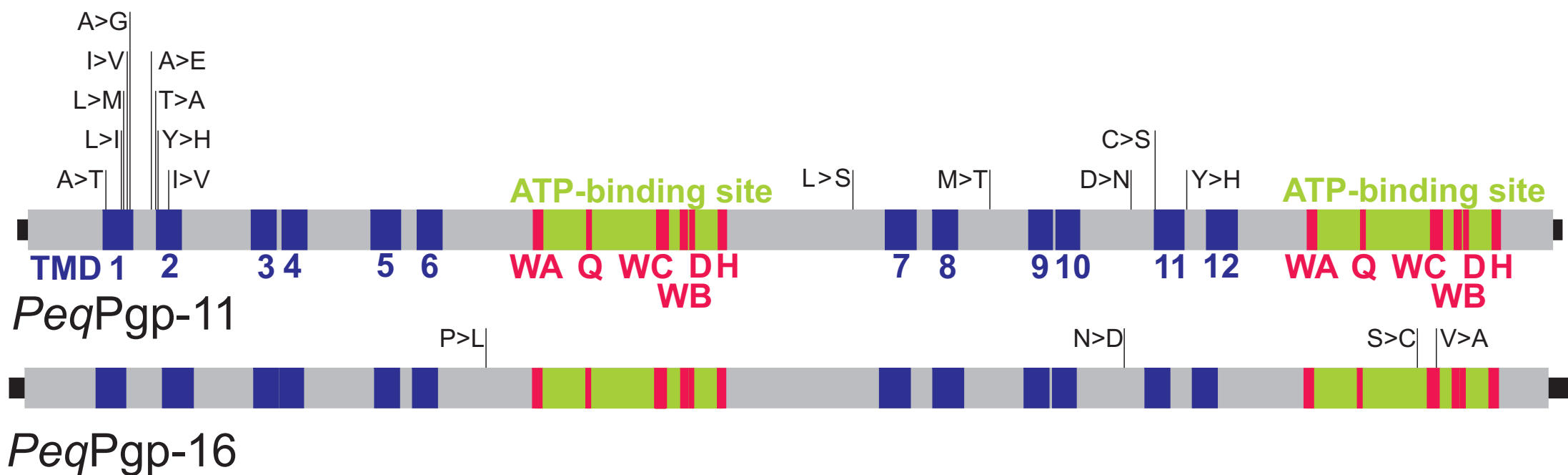

Supplement: Figure S1 — Organization of the conserved domains and identified SNPs in Peq Pgp-11 and Peq Pgp-16. ATP-binding sites are indicated in green, transmembrane domains in blue and further typical, conserved Pgp motifs in red. Length of the cDNA sequence is shaded in light grey. TMD, transmembrane domain; WA, Walker A/P-loop; WB, Walker B domain; WC, Walker C/linker peptide; D, D-loop; H, H-loop/switch region; Q, Q-loop/lid; (PDF) [file pone.0061635.s001.pdf]

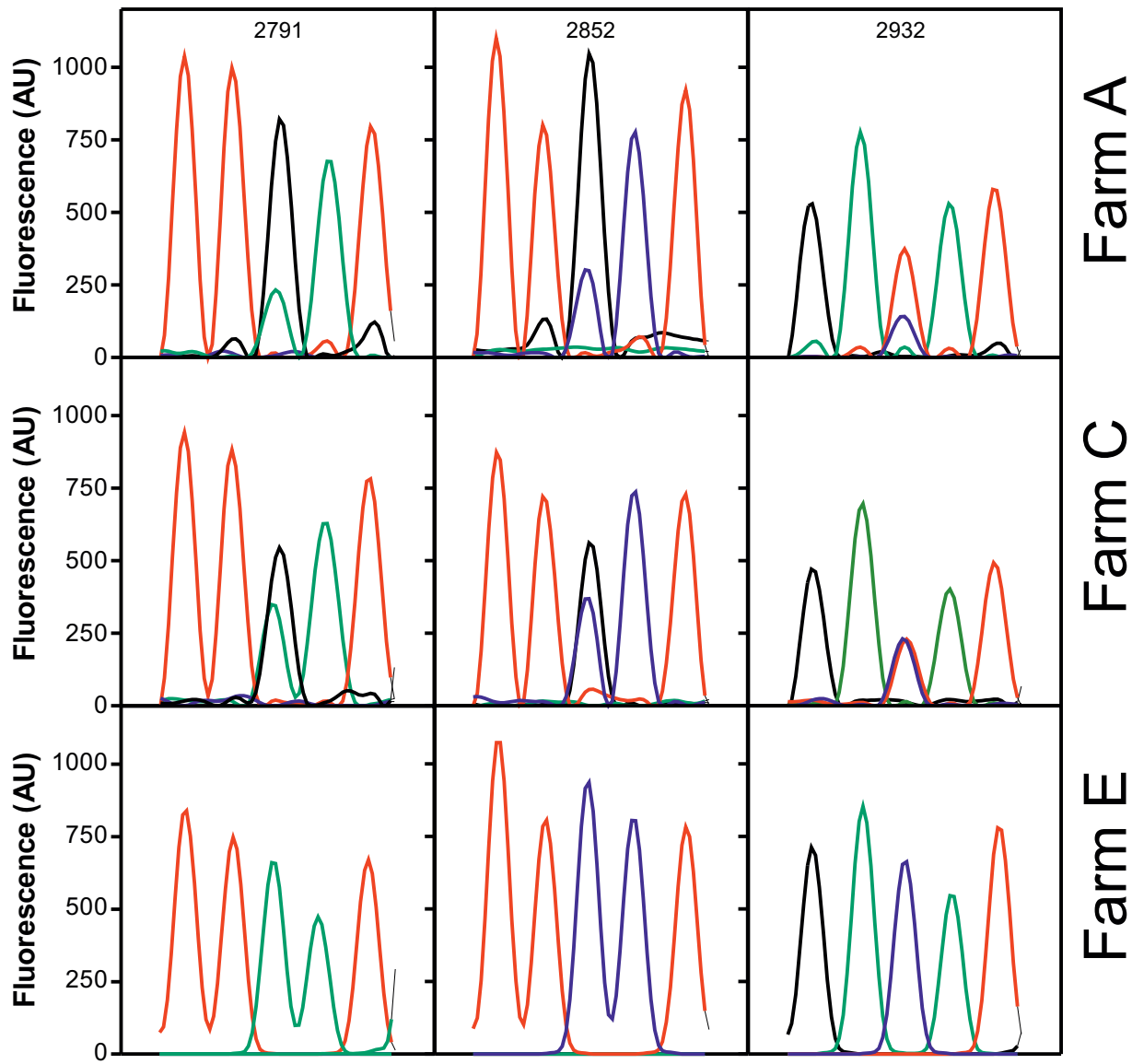

Supplement: Figure S2 — Frequencies of alleles correlating with IVM susceptibility. Chromatograms showing partial coding sequences of PeqPgp-11 with positions of ML resistance associated SNPs of three farms of different ML susceptibilities: ML susceptible (farm A), intermediate ML susceptibility (farm C) and decreased ML susceptibility (farm E). Compared to the susceptible farms, farms of intermediate susceptibility had a tendency to the resistant genotype but still both alleles were present at all positions. In contrast, bases were replaced completely in farms of decreased susceptibility. (PDF) [file pone.0061635.s002.pdf]
