# Supplementary material for: Genetic Variants and Increased Expression of Parascaris equorum P-glycoprotein-11 in Populations with Decreased Ivermectin Susceptibility
Source: PLoS One. 2013 Apr 24;8(4):e61635. doi: 10.1371/journal.pone.0061635 (PMC3634834; doi:10.1371/journal.pone.0061635)
Supplement: Figure S3 — Three dimensional model of Peq Pgp-11 structure. (A) Residues corresponding to drug binding residues in the mouse Pgp-1 [40] (blue) and residues differing in PeqPgp-11 due to the three SNPs correlating with decreased IVM susceptibility (red) are highlighted in the 3D model. (B) Partial sequence alignments of mouse Pgp-1 and PeqPgp-11 for regions involved in substrate binding. Identical and similar residues which are highlighted in dark grey and light grey, respectively. Amino acids known to be involved in substrate binding in M. musculus Pgp-1 are marked by asterisks. (PDF) [file pone.0061635.s003.pdf]

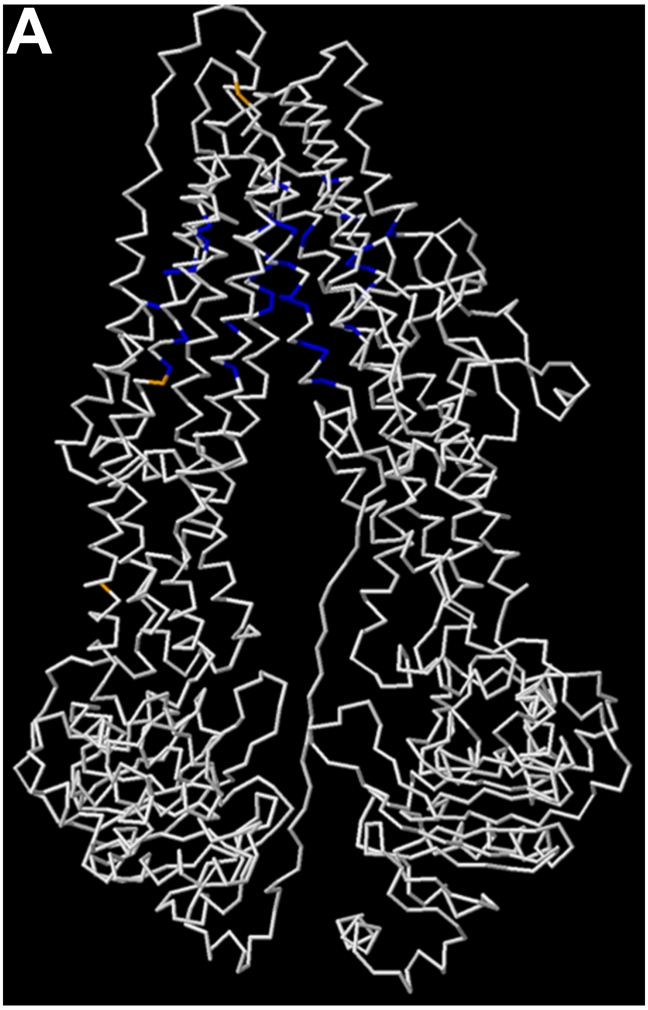

B

```
                                                        *  **   *
MmuPgp         24   KEKKEKKPAVSVLTMFRYAGWLDRLYMLVGTLAAIIHGVALPLMMLIFGDMTDSFASVGN     83
PeqPgp-11      40   KQKLEAKP-VSFFELFRFASTFDKFLVACGILLALLCGIGLPLTTILAGRLTNAL-----     93

                                    *
MmuPgp        202   IGFTRGWKLTLVILAISPVLGLSAGIWAKILSSFTDKELHAYAKAGAVAEEVLAAIRTVI    261
PeqPgp-11     204   IAFAFEWRITLVLIGLAPLSAVLMSLSSSLIDKASTKQMKHNAECAAILEESVMNFKTIA    263

                                                          * **
MmuPgp        262   AFGGQKKELERYNNNLEEAKRLGIKKAITANISMGAAFLLIYASYALAFWYGTSLV-ISK    320
PeqPgp-11     264   SCNGQQTVLKKYANGLRMARKYATQIAAFSGLFDGIFYCAIYVFFAAGFYYGGYLYKVGA    323

                               *  ** **
MmuPgp        321   EYSIGQVLTVFFSVLIGAFSVGQASPNIEAFA
PeqPgp-11     324   VTEPGGVFIVSNAVVFGAYFLGILSPHLMAVT

                          *  *   *                              *
MmuPgp        715   IINGGLQPAFSVIFSKVVGVFTNGGPPETQRQNSNLFSLL-FLILGIISFITFFLQGFTF    773
PeqPgp-11     731   VIRGAEIPLFVVIFKITFDGFVAADHDTMMKW--LLYSLISFIALGVFLLFVMFLANVFF    788

                                                                               *
MmuPgp        774   GKAGEILTKRLRYMVFKSMLRQDVSWFDDPKNTTGALTTRLANDAAQVKGATGSRLAVIF    833
PeqPgp-11     789   GWTGECVVDSLRFRALSNMLHQDAAYFDTPSRSTAITVTRLSTDAPNIKGALDVRMVQIV    848

                                                  *   *
MmuPgp        834   QNIANLGTGIIISLIYGWQLTLLLLAIVPIIAIAGVVEMKMLSGQALKDKKELEGSGKIA    893
PeqPgp-11     849   NNLVALAVTLVLGIAYCWQVGLLGLGFTLLVLFLLIVVSKFMDKTNDTAIRE-DFTGQLS    907

                                                                *  *       *
MmuPgp        894   TEAIENFRTVVSLTREQKFETMYAQSLQIPYRNAMKKAHVFGITFSFTQAMMYFSYAACF    953
PeqPgp-11     908   IEIVEQVRTIQLLTREKHFCKRFNGKLDAALVLQKKCGPPEAVSFTITMAFPFFADMVTY    967

                                     *  **  * ***  **  *
MmuPgp        954   RFGAYLVTQQLMTFENVLLVFSAIVFGAMAVGQVSSFAPDYAKATVSASHIIRIIEKTPE   1013
PeqPgp-11     968   ALGISLLYYGHAKADEVFASAMAPNSAGWAIVLLSGCLNTFFAASTSVDSVLRLV-YAPK   1026
```
