# Supplementary material for: Genetic Variants and Increased Expression of Parascaris equorum P-glycoprotein-11 in Populations with Decreased Ivermectin Susceptibility
Source: PLoS One. 2013 Apr 24;8(4):e61635. doi: 10.1371/journal.pone.0061635 (PMC3634834; doi:10.1371/journal.pone.0061635)
Supplement: Table S1 — Primer sets used for RACE PCR and amplification of two full length sequences of P. equorum Pgps. (DOCX) [file pone.0061635.s004.docx]

| **Gene** | **Primer** | **Sequence 5'- 3'** | **Annealing temperature (°C)** |
| --- | --- | --- | --- |
| *Peq*Pgp-11 | *Peq*Pgp-11-5RACE-SP1 | ttg gta agg cag acg cga cgg atg agg aga t | 55 °C |
|  | *Peq*Pgp-11-5RACE-SP2 | ttg gag ttg tgc agc agg aac cag aat tgt tca a | 59 °C |
|  | *Peq*Pgp-11-5RACE-SP3 | tac gag tgc aca gga ggg aca gta tcg atc gac gg | 60 °C |
|  | *Peq*Pgp-11-3RACE-SP5 | cag cag gaa cca gaa ttg ttc aat ggg acg atc aa |  |
|  | *Peq*Pgp-11-3RACE-SP6 | gat cat tga gta ctg tca aat ggc caa cgc gca c |  |
|  | *Peq*Pgp-11-full-length-Sense  *Peq*Pgp-11-full-length-ASense | ccc aag ttt gag gca atg tc  tca gta cca ggc gta gtt cg | 55 °C |
|  |  |  |  |
| *Peq*Pgp-16 | *Peq*Pgp-16-5RACE-SP1 | aag ata tga ccg acg agg aaa tgg aac g | 55 °C |
|  | *Peq*Pgp-16-5RACE-SP2 | aat acc att ggt gtt gtc tct cag gaa cc tgta ct | 60 °C |
|  | *Peq*Pgp-16-5RACE-SP3 | atg gca ttc caa tca aag aac tga acc tag gat g |  |
|  | *Peq*Pgp-16-3RACE-SP5 | ccg tct tgg taa aga aga tat gac cga cga gga a |  |
|  | *Peq*Pgp-16-3RACE-SP6 | gtg ttt gta aaa tgg cga atg cac aca att tca t |  |
|  | *Peq*Pgp-16-full-length-Sense  *Peq*Pgp-16-full-length-ASense | cga cac act tcg tgc tta aca g  tgg att tca taa acg aga aag ata | 55 °C |

**Table S1.** **Primer sets used for RACE PCR and amplification of two full length sequences of**

***P. equorum* Pgps.**
