# Supplementary material for: Genetic Variants and Increased Expression of Parascaris equorum P-glycoprotein-11 in Populations with Decreased Ivermectin Susceptibility
Source: PLoS One. 2013 Apr 24;8(4):e61635. doi: 10.1371/journal.pone.0061635 (PMC3634834; doi:10.1371/journal.pone.0061635)
Supplement: Table S2 — Primer sets used for amplification of PCR products compared by SeqDoC analysis. (DOCX) [file pone.0061635.s005.docx]

**Table S2.** **Primer sets used for amplification of PCR products compared by SeqDoC analysis.**

| **Gene** | **Primer** | **Sequence 5'-3'** | **Amplicon size (bp)** |
| --- | --- | --- | --- |
| Pgp-11 | Pgp-11-SeqDoC-1-F  Pgp-11-SeqDoC-1-R | ccc aag ttt gag gca atg tc  tgg aga taa agt tgg gct ca | 596 |
|  | Pgp-11-SeqDoC-2-F  Pgp-11-SeqDoC-2-R | gaa gtt tac tgc gac agg  cgt gaa atc tgg cga tac | 693 |
|  | Pgp-11-SeqDoC-3-F  Pgp-11-SeqDoC-3-R | gtc atc gga aga ggg cat t  atg ctg aaa gcg aaa tca cc | 573 |
|  | Pgp-11-SeqDoC-4-F  Pgp-11-SeqDoC-4-R | cgg ata ttg ctg ttg gat ga  gta att cgt ggt gcc gag at | 565 |
|  | Pgp-11-SeqDoC-5-F  Pgp-11-SeqDoC-5-R | gcg agg tct gtg gca ttt at  ggt act gcg atc agg gaa ga | 584 |
|  | Pgp-11-SeqDoC-6-F  Pgp-11-SeqDoC-6-R | ggt ttc acg ctc ctt gtt ct  ctc agg gca tct aag ggt ca | 598 |
|  | Pgp-11-SeqDoC-7-F  Pgp-11-SeqDoC-7-R | cgc ttc tct tat ccg acc ag  ttc ttc gat tca aca cgc ag | 594 |
|  | Pgp-11-SeqDoC-8-F  Pgp-11-SeqDoC-8-R | gcg gta cga ttc gtg aaa at  tca gta cca ggc gta gtt cg | 501 |
|  |  |  |  |
| Pgp-16 | Pgp-16-SeqDoC-1-F  Pgp-16-SeqDoC-1-R | cga cac act tcg tgc tta aca g  tca ggc gat tta acg aca aa | 560 |
|  | Pgp-16-SeqDoC-2A-F  Pgp-16-SeqDoC-2A-R | att ttc gca ctc gct ttc at  taa agc agg tgc aat tgc tg | 390 |
|  | Pgp-16-SeqDoC-2B-F  Pgp-16-SeqDoC-2B-R | ggg tgg act tat tgg cct tc  tat ggg agg tac cgt ggc ta | 358 |
|  | Pgp-16-SeqDoC-3-F  Pgp-16-SeqDoC-3-R | aat ggg tgt tgc att ttg gt  gac gag gaa atg gaa cgt gt | 581 |
|  | Pgp-16-SeqDoC-4-F  Pgp-16-SeqDoC-4-R | tgg tgt tgt ctc tca gga acc  gcc tat aaa tcg atc gcg aa | 586 |
|  | Pgp-16-SeqDoC-5-F  Pgp-16-SeqDoC-5-R | cat aga tga gtt gcg cga taa  ctt aca act cgt ctt gcc acc | 555 |
|  | Pgp-16-SeqDoC-6-F  Pgp-16-SeqDoC-6-R | gac gtc tcg tct tcg ttt ga  atc gaa gca ttg aac tgt gc | 599 |
|  | Pgp-16-SeqDoC-7-F  Pgp-16-SeqDoC-7-R | tcg cat gtg ctt atc gtt tc  aga ggc tgc caa act tgc ta | 590 |
|  | Pgp-16-SeqDoC-8-F  Pgp-16-SeqDoC-8-R | gct cag gcg gta aat ttg ag  tgg att tca taa acg aga aag ata | 586 |
