# Supplementary material for: Genetic Variants and Increased Expression of Parascaris equorum P-glycoprotein-11 in Populations with Decreased Ivermectin Susceptibility
Source: PLoS One. 2013 Apr 24;8(4):e61635. doi: 10.1371/journal.pone.0061635 (PMC3634834; doi:10.1371/journal.pone.0061635)
Supplement: Table S3 — Primer pairs used for real-time PCR of Peq Pgp-11, Peq Pgp-16 and reference genes actin, gpd-1 and 18SrRNA. (DOCX) [file pone.0061635.s006.docx]

| **Gene** | **Accession number** | **Primer** | **Sequence 5'-3'** | **Amplicon size (bp)** |
| --- | --- | --- | --- | --- |
| Pgp-11 | JX308230 | *Peq*Pgp-11-qPCR-F  *Peq*Pgp-11-qPCR-R | gtc atc gga aga ggg cat t  gtg aaa tct ggc gat acg gt | 137 |
| Pgp-16 | JX308231 | *Peq*Pgp-16-qPCR-F  *Peq*Pgp-16-qPCR-R | gaa aag cga caa cat cac ga  gag cat aga gtg gag ccg tc | 120 |
| Actin | unpublished data | *Peq*-act1-qPCR -F  *Peq*-act1-qPCR -R | tcg ttt tta ggg gag gga tg  aaa cac cga gca aaa tgg ag | 137 |
| gpd-1 | unpublished data | *Peq*-gpd1-qPCR -F  *Peq*-gpd1-qPCR -R | atc ggt tgt cga tct tac gg  gac ttt gtt ggt gat tcg ca | 152 |
| 18SrRNA | [U94378](http://www.ncbi.nlm.nih.gov/nucleotide/2772635?report=genbank&log$=nucltop&blast_rank=1&RID=NED2CZ6Z016) | *Peq*-18SrRNA -qPCR-F  *Peq*-18SrRNA -qPCR-R | atg gcc gtt ctt agt tgg tg  tac agc cgc atg aag ttg ag | 141 |

**Table S3.** **Primer pairs used for real-time PCR of *Peq*Pgp-11, *Peq*Pgp-16 and reference genes actin, gpd-1 and 18SrRNA.**
