# Supplementary material for: Genetic Variants and Increased Expression of Parascaris equorum P-glycoprotein-11 in Populations with Decreased Ivermectin Susceptibility
Source: PLoS One. 2013 Apr 24;8(4):e61635. doi: 10.1371/journal.pone.0061635 (PMC3634834; doi:10.1371/journal.pone.0061635)
Supplement: Table S4 — Primer set for gender determination. Degenerated primers were generated from corresponding sequences of Ascaris suum , C. elegans and C. briggsae . (DOCX) [file pone.0061635.s007.docx]

| **Gene** | **Primer** | **Sequence 5'- 3'** | **Annealing Temperature (°C)** | **Amplicon size (bp)** |
| --- | --- | --- | --- | --- |
| Vitellogenin-6 | Peq-Vit-6-F  Peq-Vit-6-R | cgm tay gag aag gtb ctt gcy ttg aag acm c  tgc cwc gca aga tcc aac adv ttc tbm tsc | 56 | 228 |

**Table S4.** **Primer set for gender determination. Degenerated primers were generated from corresponding sequences of *Ascaris suum*, *C. elegans* and *C. briggsae*.**
