# Supplementary material for: Genetic Variants and Increased Expression of Parascaris equorum P-glycoprotein-11 in Populations with Decreased Ivermectin Susceptibility
Source: PLoS One. 2013 Apr 24;8(4):e61635. doi: 10.1371/journal.pone.0061635 (PMC3634834; doi:10.1371/journal.pone.0061635)
Supplement: Table S5 — NCBI accession numbers and Worm Base ID for protein sequences used for maximum likelihood tree. (DOCX) [file pone.0061635.s008.docx]

**Table S5 NCBI accession numbers and Worm Base ID for protein sequences used for maximum likelihood tree.**

| **Name** | **Organism** | **GenBank accession no.** | **WormBase ID** |
| --- | --- | --- | --- |
| *Asu*MRP-1 | *Ascaris suum* | ADY40620 |  |
| *Asu*MRP-3a | *Ascaris suum* | ADY40644 |  |
| *Asu*MRP-3b | *Ascaris suum* | ADY40573 |  |
| *Bma*MRP-3 | *Brugia malayi* | XP_001896434 |  |
| *Bma*Pgp-1 | *Brugia malayi* | XP_001900095 |  |
| *Bma*Pgp-2 | *Brugia malayi* | XP_001897744 |  |
| *Cbr*Pgp-1 | *Caenorhabditis briggsae* | XP_002634978 | BP:CBP17817 |
| *Cbr*Pgp-2 | *Caenorhabditis briggsae* | XP_002639421 | BP:CBP33028 |
| *Cbr*Pgp-3 | *Caenorhabditis briggsae* | XP_002643979 | BP:CBP26138 |
| *Cbr*Pgp-4 | *Caenorhabditis briggsae* | XP_002643978 | BP:CBP2677 |
| *Cbr*Pgp-7 | *Caenorhabditis briggsae* | XP_002644094 | BP:CBP25824 |
| *Cbr*Pgp-8 | *Caenorhabditis briggsae* | XP_002644095 | BP:CBP34515 |
| *Cbr*Pgp-9 | *Caenorhabditis briggsae* | XP_002638613 | BP:CBP25885 |
| *Cbr*Pgp-10 | *Caenorhabditis briggsae* | XP_003117716 | BP:CBP25360 |
| *Cbr*Pgp-11 | *Caenorhabditis briggsae* | XP_002629994 | BP:CBP09377 |
| *Cbr*Pgp-12 | *Caenorhabditis briggsae* | XP_002645224 | BP:CBP35051 |
| *Cbr*Pgp-13 | *Caenorhabditis briggsae* | XP_002645222 | BP:CBP27772 |
| *Cbr*Pgp-14 | *Caenorhabditis briggsae* | XP_002645220 | BP:CBP35857 |
| *Cbr*Pgp-CBG12969 | *Caenorhabditis briggsae* | XP_002630530 | BP:CBP17602 |
| *Cel*Pgp-1 | *Caenorhabditis elegans* | NP_502413 | WP:CE11932 |
| *Cel*Pgp-2 | *Caenorhabditis elegans* | NP_491707 | WP:CE41207 |
| *Cel*Pgp-3 | *Caenorhabditis elegans* | NP_509901 | WP:CE03818 |
| *Cel*Pgp-4 | *Caenorhabditis elegans* | NP_001257143 | WP:CE03308 |
| *Cel*Pgp-5 | *Caenorhabditis elegans* | NP_001257116 | WP:CE43003 |
| *Cel*Pgp-6 | *Caenorhabditis elegans* | NP_001041287 | WP:CE40818 |
| *Cel*Pgp-7 | *Caenorhabditis elegans* | NP_509812 | WP:CE36668 |
| *Cel*Pgp-8 | *Caenorhabditis elegans* | NP_509811 | WP:CE31624 |
| *Cel*Pgp-9 | *Caenorhabditis elegans* | NP_507487 | WP:CE15714 |
| *Cel*Pgp-10 | *Caenorhabditis elegans* | NP_509205 | WP:CE40807 |
| *Cel*Pgp-11 | *Caenorhabditis elegans* | NP_495674 | WP:CE34788 |
| *Cel*Pgp-12 | *Caenorhabditis elegans* | NP_510126 | WP:CE03260 |
| *Cel*Pgp-13 | *Caenorhabditis elegans* | NP_510127 | WP:CE40253 |
| *Cel*Pgp-14 | *Caenorhabditis elegans* | NP_510128 | WP:CE0262 |
| *Dme*MDR-50 | *Drosophila melanogaster* | AAA16186 |  |
| *Dme*MDR-49 | *Drosophila melanogaster* | AAA28679 |  |
| *Dme*MDR-65 | *Drosophila melanogaster* | AAA28680 |  |
| *Hco*Pgp-2 | *Haemonchus contortus* | AAC38987 |  |
| *Mmu*ABCB-1b | *Mus musculus* | NP_035205 |  |
| *Mmu*Pgp | *Mus musculus* | AAA39514 |  |
| *Mca*Pgp-L | *Mytilus californianus* | ABS83556 |  |
| *Mga*Pgp-L | *Mytilus galloprovincialis* | CAX46411 |  |
| *Ovo*Pgp-1 | *Onchocerca volvulus* | AAD49436 |  |
| *Ovo*Pgp | *Onchocerca volvulus* | AAX82635 |  |
| *Ovo*Pgp-L | *Onchocerca volvulus* | AAD49563 |  |
| *Peq*Pgp-11 | *Parascaris equorum* | JX308230 |  |
| *Peq*Pgp-16 | *Parascaris equorum* | JX308231 |  |
| *Phu*MRP-1 | *Pediculus humanus corporis* | XP_002425149 |  |
| *Phu*MRP-2 | *Pediculus humanus corporis* | XP_002432260 |  |
| *Phu*MRP-3 | *Pediculus humanus corporis* | XP_002426586 |  |
| *Phu*MRP-4 | *Pediculus humanus corporis* | XP_002425021 |  |
| *Ppa*Pgp-1 | *Pristionchus pacificus* |  | PP:PP30697 |
| *Ppa*Pgp-9 | *Pristionchus pacificus* |  | PP:PP30465 |
| *Sma*Pgp-1,2,3 | *Schistosoma mansoni* | XP_002574196 |  |
