# Supplementary material for: Genetic Variants and Increased Expression of Parascaris equorum P-glycoprotein-11 in Populations with Decreased Ivermectin Susceptibility
Source: PLoS One. 2013 Apr 24;8(4):e61635. doi: 10.1371/journal.pone.0061635 (PMC3634834; doi:10.1371/journal.pone.0061635)
Supplement: Table S6 — Position of single-nucleotide-polymorphisms in Peq Pgp-11 detected by SeqDoC analysis. (DOCX) [file pone.0061635.s009.docx]

**Table S6. Position of single-nucleotide-polymorphisms in *Peq*Pgp-11 detected by SeqDoC analysis.**

| **Position in full-length-sequence of**  ***Peq*Pgp-11** | **Base-exchange** | **Amino acid** | **Chemical distance of amino acid change^a^** |
| --- | --- | --- | --- |
| 196 | G 🡪 A | Ala 🡪 Thr | 58 |
| 210 | G 🡪 A | Leu | - |
| 235 | C 🡪 A | Leu 🡪 Ile | 5 |
| 241 | T 🡪 A | Leu 🡪 Met | 15 |
| 250 | A 🡪 G | Ile 🡪 Val | 29 |
| 257 | C 🡪 G | Ala 🡪 Gly | 60 |
| 311 | C 🡪 A | Ala 🡪 Glu | 107 |
| 322 | A 🡪 G | Thr 🡪 Ala | 58 |
| 327 | A 🡪 G | Gly | - |
| 328 | T 🡪 C | Tyr 🡪 His | 83 |
| 355 | A 🡪 G | Ile 🡪 Val | 29 |
| 468 | G 🡪 A | Gln | - |
| 774 | C 🡪 T | Asn | - |
| 1069 | G 🡪 T | Ala | - |
| 1309 | G 🡪 A | Gly/Ser 🡪 Ser | - |
| 1363 | G 🡪 A | Val | - |
| 1392 | A 🡪 T | Ser | - |
| 1479 | T 🡪 C | Asp | - |
| 1527 | T 🡪 C | His | - |
| 2087 | T 🡪 C | Leu 🡪 Ser | 145 |
| 2433 | T 🡪 C | Met 🡪 Thr | 81 |
| 2586 | T 🡪 C | Ile | - |
| 2645 | A 🡪 T | Leu/His 🡪 Leu | - |
| 2791 | G 🡪 A | Asp 🡪 Asn | 23 |
| 2852 | G 🡪 C | Cys 🡪 Ser | 112 |
| 2874 | T 🡪 C | Phe | - |
| 2905 | T 🡪 C | Leu/Phe 🡪 Leu | - |
| 2932 | T 🡪 C | Tyr 🡪 His | 83 |
| 3855 | A 🡪 G | Ser | - |
