# Supplementary material for: Genetic Variants and Increased Expression of Parascaris equorum P-glycoprotein-11 in Populations with Decreased Ivermectin Susceptibility
Source: PLoS One. 2013 Apr 24;8(4):e61635. doi: 10.1371/journal.pone.0061635 (PMC3634834; doi:10.1371/journal.pone.0061635)
Supplement: Table S7 — Position of single-nucleotide-polymorphisms in Peq Pgp-16 detected by SeqDoC analysis. (DOCX) [file pone.0061635.s010.docx]

**Table S7.** **Position of single-nucleotide-polymorphisms in *Peq*Pgp-16 detected by SeqDoC analysis.**

| **Position in full-length-sequence of**  ***Peq*Pgp-16** | **Base** | **Amino acid** | **Chemical distance of amino acid change^a^** |
| --- | --- | --- | --- |
| 1166 | C 🡪 T | Pro 🡪 Leu | 98 |
| 1239 | T 🡪 C | Asp | - |
| 2280 | A 🡪 C | Ala | - |
| 2782 | A 🡪 G | Asn 🡪 Asp | 23 |
| 2904 | T 🡪 C | Arg | - |
| 3524 | G 🡪 C | Ser 🡪 Cys | 112 |
| 3572 | T 🡪 C | Val 🡪 Ala | 64 |

^a^Grantham R (1974) Amino acid difference formula to help explain protein evolution. Science 185: 862-864.
